# Regulating the dormancy of cancer stem cells: a novel approach to preventing cancer relapse

**DOI:** 10.1038/s41419-026-08707-z

**Published:** 2026-04-04

**Authors:** Qian Wang, Ning Liang, Xiongchao Fang, Tao Yang, Xianli He, Gang Wang, Nan Wang

**Affiliations:** 1 Department of General Surgery, Tangdu Hospital, Fourth Military Medical University, Xi’an, China; 2 Department of Gastroenterology, 920th Hospital of the Joint Logistics Support Force, PLA, Kunming, China; 3 Department of Interventional Radiology, Tangdu Hospital, Fourth Military Medical University, Xi’an, China

**Keywords:** Cancer stem cells, Quiescence, Cancer stem cells

## Abstract

Dormant cancer stem cells (CSCs) are the root cause of the drug resistance and metastatic processes of malignant tumors, but an in-depth analysis of their biological mechanisms is needed. Dormant CSCs are in the G0 phase of the cell cycle and are characterized by enhanced autophagic activity, a stable genomic structure and strong plasticity. Recently, several new specific markers of dormant CSCs, such as p27, CD13, QSOX1, Survivin, GPD1 and BEX2, have been identified, which offer hope for targeted therapy. In addition, epigenetic modifications such as DNA methylation and histone modifications have been reported to regulate the transition between the quiescent and proliferative states of dormant CSCs. From a clinical perspective, keeping cancer stem cells in a dormant state is helpful for preventing tumor recurrence and metastasis. To this end, clarifying the potential mechanisms and molecular regulation of cancer stem cell dormancy is vital. Here, in this review, we examine recent significant findings regarding tumor stem cell dormancy in both experimental and human disease models, emphasizing the underlying molecular mechanisms, regulatory processes, experimental models, and prospective research directions aimed at advancing this field and enhancing clinical translation.

## Facts

- The dormancy mechanism of tumor cells has been widely studied, but the dormancy mechanism of cancer stem cells (CSCs) has not been fully elucidated.
- Chemotherapy can awaken dormant cancer stem cells (dormant CSCs), thereby promoting tumor recurrence and metastasis.
- Dormant CSCs closely communicate with fibroblasts, mesenchymal stem cells and immune cells in the tumor microenvironment.
- Keeping CSCs in a dormant state may help prevent the recurrence and metastasis of tumors.

## Open Questions

- What is the mechanism by which cancer stem cells maintain dormancy?
- How can the resistance of dormant CSCs to chemotherapy, radiotherapy, and targeted drugs be overcome?
- How can real-time tracking technology for dormant CSCs be developed to facilitate therapeutic effect evaluation?

## Introduction

**Fig. 1:**
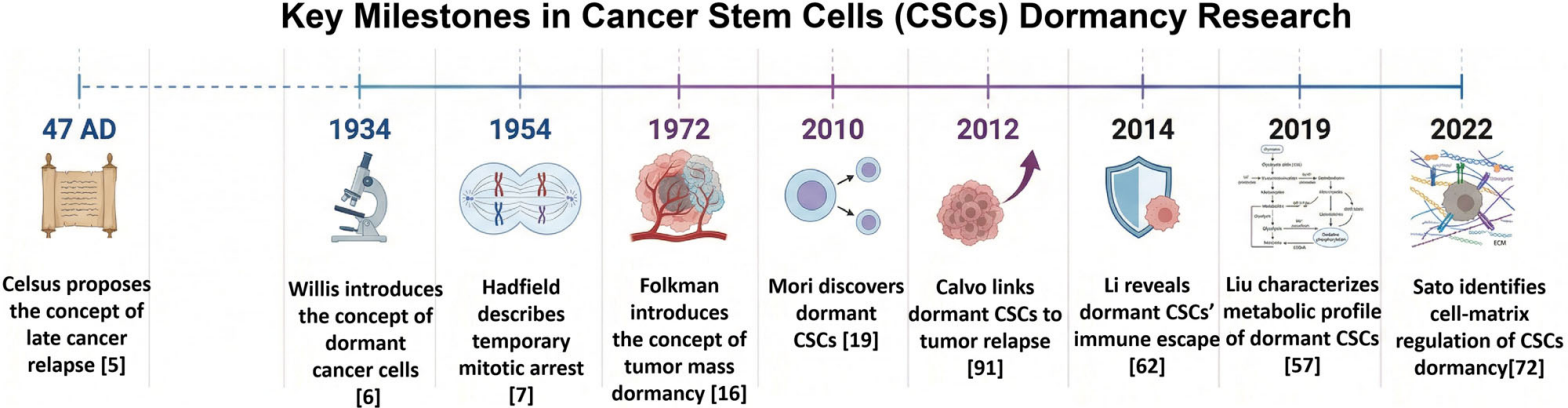
Brief history of cancer stem cell dormancy. A timeline illustrating the origins of the dormancy concept and the development of the concepts of cancer cell dormancy [7] and tumor mass dormancy [16]. In 2010, the concept of dormant CSCs was proposed [19]. More recently, the metabolic characteristics of dormant CSCs [57] and their role in immune escape have been studied [62].

Metastasis is the leading cause of cancer death [1–4]. The concept of cancer recurrence can be traced back to the first century, as evidenced by Celsus’s first observation that “even if a scar is formed after excision, the disease will still recur and lead to death” (Fig. 1) [5]. In 1934, Rupert Willis discovered that distant metastases could occur in patients without local recurrence, suggesting that tumor cells might remain dormant in metastatic tissue[6]. In 1954, Jeffrey Hadfield advanced the concept that dormant cancer cells in distant metastases undergo a phase of “temporary mitotic arrest” [7]. In fact, cancer cell dormancy is a recognized phenomenon in many common solid cancers, including breast, lung, colon, melanoma, and leukemia [8–13]. Clinically, more than 25% of breast cancer patients experience long-term recurrence following treatment, a phenomenon referred to as dormant metastasis [14, 15]. In 1972, Judah Folkman introduced the concept of “tumor mass dormancy”, which describes a state in which a tumor remains at a low but undetectable steady-state level because of a balance between cell growth and death. Notably, the concept of “tumor mass dormancy” is significantly different from that of “dormant cancer cells”, as the latter do not proliferate [16].

**Fig. 2:**
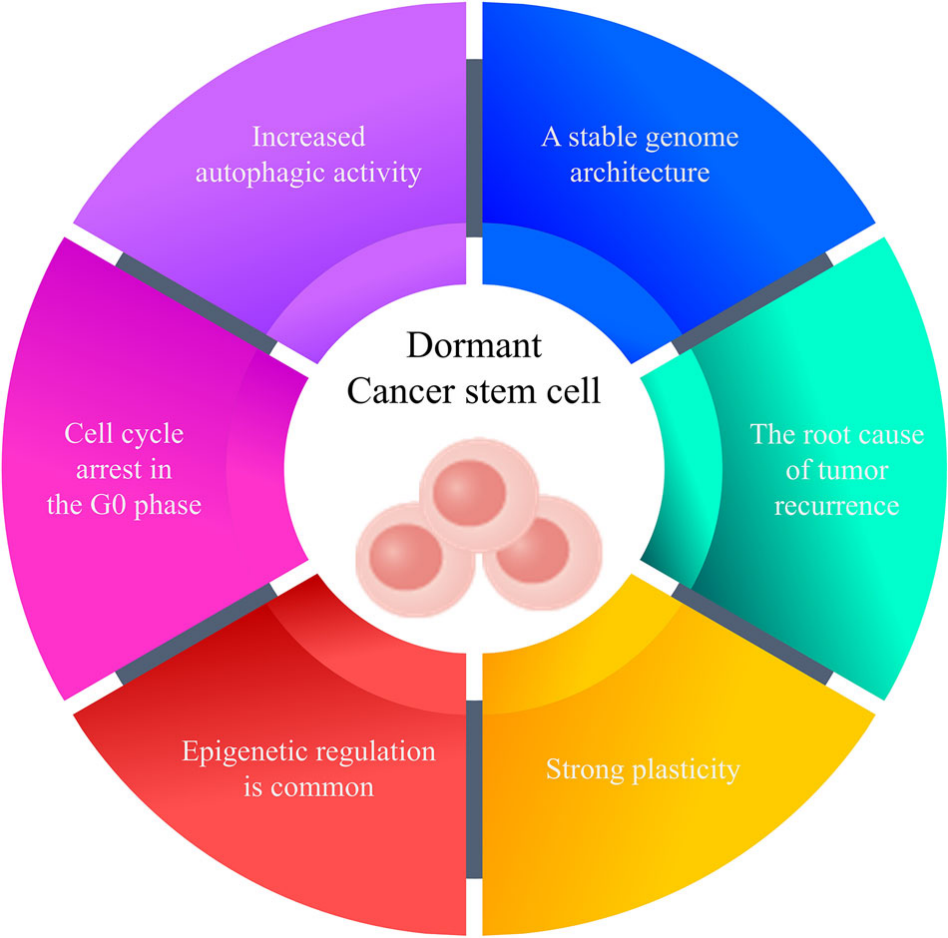
Biological characteristics of dormant tumor stem cells. Dormant tumor stem cells have the following six biological characteristics: (1) cells in the G0 phase; (2) enhanced autophagic activity; (3) a stable genome structure; (4) strong plasticity; (5) subject to epigenetic regulation; and (6) the ability to cause tumor recurrence.

Cancer stem cells (CSCs) are a small fraction of cells present in tumor tissue that can self-renew and produce heterogeneous tumor cells [17, 18]. In 2010, Masaki Mori proposed the concept of dormant CSCs, which represent a distinct subset of CSCs characterized by their ability to enter a reversible state of cell cycle arrest [19]. To date, dormant CSCs have been found in a variety of cancer tissues, including colorectal cancer, liver cancer, leukemia, esophageal cancer, breast cancer, and gastric cancer [20, 21]. Dormant CSCs do not proliferate and have low metabolic activity but maintain intact stem cell characteristics (self-renewal and differentiation potential) [22]. Dormant CSCs can produce progeny and differentiated cells through asymmetric division to sustain tumor growth [22]. Additionally, dormant CSCs have the potential for multilineage differentiation, allowing them to differentiate into various subtypes of tumor cells that contribute to increased tumor heterogeneity [23]. Dormant CSCs are the root cause of recurrence and metastasis of malignant tumors [24]. The dormancy of CSCs is a unique biological event characterized by arrest of the cell cycle at the G0 phase (Fig. 2) [25]. This dormant state helps CSCs survive under unfavorable conditions, such as nutrient shortage or low oxygen, and helps them evade attacks from the host immune system [25]. When environmental conditions improve, they can proliferate again and cause tumor recurrence [26]. Dormant CSCs are the most “cunning” and “stubborn” type of CSCs. All dormant CSCs are CSCs, but not all CSCs are in a dormant state. CSCs can dynamically switch between an active and dormant on the basis of microenvironmental signals. This plasticity is among the key reasons why cancer is difficult to cure. In summary, CSCs are the “engines” of tumors, while dormant CSCs are deeply hidden and equipped with a “delayed start” backup engine. One of the main challenges in current cancer treatment is how to find and destroy these backup engines. The differences and similarities between CSCs and dormant CSCs are summarized in Table 1.

**Table 1:** Differences and similarities between CSCs and dormant CSCs.

Characteristic	Cancer stem cells (CSCs)	Dormant cancer stem cells (dormant CSCs)
Cell cycle state	Slow proliferation or preparation for proliferation	Completely static/dormant state (G0 phase)
Role in tumors	Drive the sustained growth and maintenance of primary tumors	The main cause of long-term recurrence and metastasis
Resistance to conventional treatment	Relative resistance (due to slow cell cycle and resistance-related proteins)	Extremely resistant (due to complete stillness, not participating in cell division at all)
Difficulty of detection	Difficult to detect, but still identifiable by specific methods due to their activity	Extremely difficult to detect and target, as they are in a quiescent state with almost no biological activity
Relationship	All dormant CSCs are CSCs, but not all CSCs are in a dormant state	A unique and the most dangerous subset of CSCs
Analogy	Active “seeds” and “roots”	The Seed of Deep Hibernation

Recent research has confirmed that a key feature of dormant CSCs is their ability to maintain genome integrity [22]. By using Green Fluorescent Protein (GFP)-labeled reverse transcription viral transfection in the Mary-X human PDX model, Barsky and colleagues reported that most tumor cells showed numerous gene amplification and deletion events, while the dormant CSC subpopulation had a stable genome architecture and distinct morphology [22]. In addition, the dormant state of dormant CSCs is reversible. For example, under low-oxygen conditions, breast CSCs undergo reversible dormancy. After the oxygen supply is restored, these cells can proliferate, indicating that dormant CSCs have strong plasticity [22, 27].

Autophagy is an evolutionarily indispensable metabolic process whose crucial role in the survival of dormant CSCs has been demonstrated by a variety of studies [28, 29]. For example, dormant ovarian CSCs exhibit high autophagic activity. Knocking out ATG5 to inhibit autophagy can lead to the death of dormant ovarian CSCs and suppress the recurrence of ovarian cancer [30]. Larrue et al. demonstrated that targeting ferritinophagy impaired quiescent acute myeloid leukemia CSCs by using a mouse patient-derived xenograft (PDX) model [31]. Baquero and colleagues reported that the second-generation autophagy inhibitor PIK-III can effectively eliminate dormant leukemia stem cells [32]. Autophagy critically helps dormant CSCs survive under stress, the core mechanisms of which may involve the following aspects. First, metabolic maintenance and energy supply. Dormant CSCs typically exist in nutrient-deficient microenvironments. Autophagy provides dormant CSCs with the energy and biosynthetic materials needed to sustain basic life activities through self-digestion, which is the foundation for their long-term survival [33, 34]. Second, with respect to the intracellular quality control system, long-term dormancy can lead to the accumulation of damaged proteins and organelles (especially mitochondria) within the cell. Autophagy, as an important quality control mechanism, removes these harmful components in a timely manner to prevent the induction of apoptosis and other types of cell death, thereby maintaining long-term homeostasis in the cells [35, 36]. Third, CSCs maintain stemness and resist therapeutic stress. Autophagy helps dormant CSCs resist cellular stress caused by treatment modalities such as chemotherapy and radiation therapy [37, 38]. These treatments typically target rapidly dividing cells but can also cause indirect damage, such as oxidative stress, to dormant cells. The activation of autophagy clears this damage and is the key to allowing dormant cells to “lurk” leading to later recurrence. Therefore, inhibiting autophagy promotes the clearance of dormant CSCs [39]. In conclusion, dormant CSCs are in the G0 phase of the cell cycle and are characterized by enhanced autophagic activity, a stable genomic structure and strong plasticity [40].

Clinically, more than 25% of patients with breast cancer experience long-term recurrence after treatment, a phenomenon known as dormant metastasis [9, 41]. Dormant CSCs are the key factors leading to metastatic dormancy in breast cancer [42]. Mechanistically, dormant breast CSCs residing in bone marrow escape the cytotoxic effects of NK cells by upregulating BACH1 and SOX2, indicating that the immune system controls dormant CSCs during disease latency [43]. Dormant CSCs are also important causes of chemotherapy resistance [44–46]. In the context of colorectal cancer, dormant CSCs increase the expression of the ABC transporter family, which activates a drug efflux mechanism that decreases the resistance barrier to chemotherapy [26]. In summary, dormant CSCs are closely related to metastatic dormancy and chemotherapy resistance [26]. In this review, we summarize the latest significant discoveries concerning the dormancy of CSCs, providing a theoretical basis and practical guidance for effectively targeting dormant CSCs.

## Relationship between dormant CSCs and tumor metastasis

**Fig. 3:**
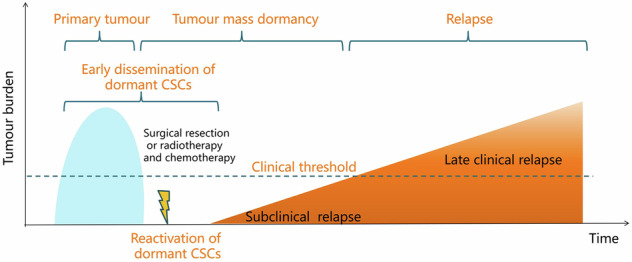
Model of cancer stem cell dormancy. During the early progression of tumors, tumor cells have already disseminated from the primary tumor to distant organs even before treatment. At a seemingly random time point, a limited number of dormant CSCs are reactivated, subsequently entering a phase of subclinical relapse. “Tumor mass dormancy” describes a state in which a tumor remains at a low but undetectable steady-state level because of a balance between cell growth and death. The subclinical relapse phase persists until the number of these cells surpasses a clinical threshold, which marks entry into a late clinical relapse phase.

Notably, the origin of dormant tumor cells that form clinical metastases is still being debated [47]. During the early progression of tumors, tumor cells are shed from the primary tumor, enter the bloodstream, and spread to distant organs (Fig. 3). These cells are called disseminated tumor cells (DTCs) [48, 49]. Most DTCs are cleared owing to the unfavorable conditions in the new environment, while a few DTCs (< 20 cells) that can enter a dormant state exist in the form of single or small clusters [29, 49]. These DTCs can then be activated by signals in the microenvironment, such as inflammation, angiogenesis, or immune changes, resulting in the formation of visible distant metastases without local relapse [50].

However, distant metastases may also be a “byproduct” of local relapse [51, 52]. Locally recurrent tumors, as new and usually more invasive lesions, can become more efficient and persistent sources of disseminated tumor cells, resulting in the delivery of new metastatic seeds throughout the body [53, 54]. In some cases, distant metastases occur soon after local relapse, supporting the view that distant metastases may also be a “byproduct” of local relapse [55, 56]. Therefore, future research should use lineage tracing techniques to accurately distinguish whether the cells in metastatic lesions in animal models originate from the primary tumors or local relapsed tumors.

## Dormancy characteristics of CSCs in different types of cancer

**Fig. 4:**
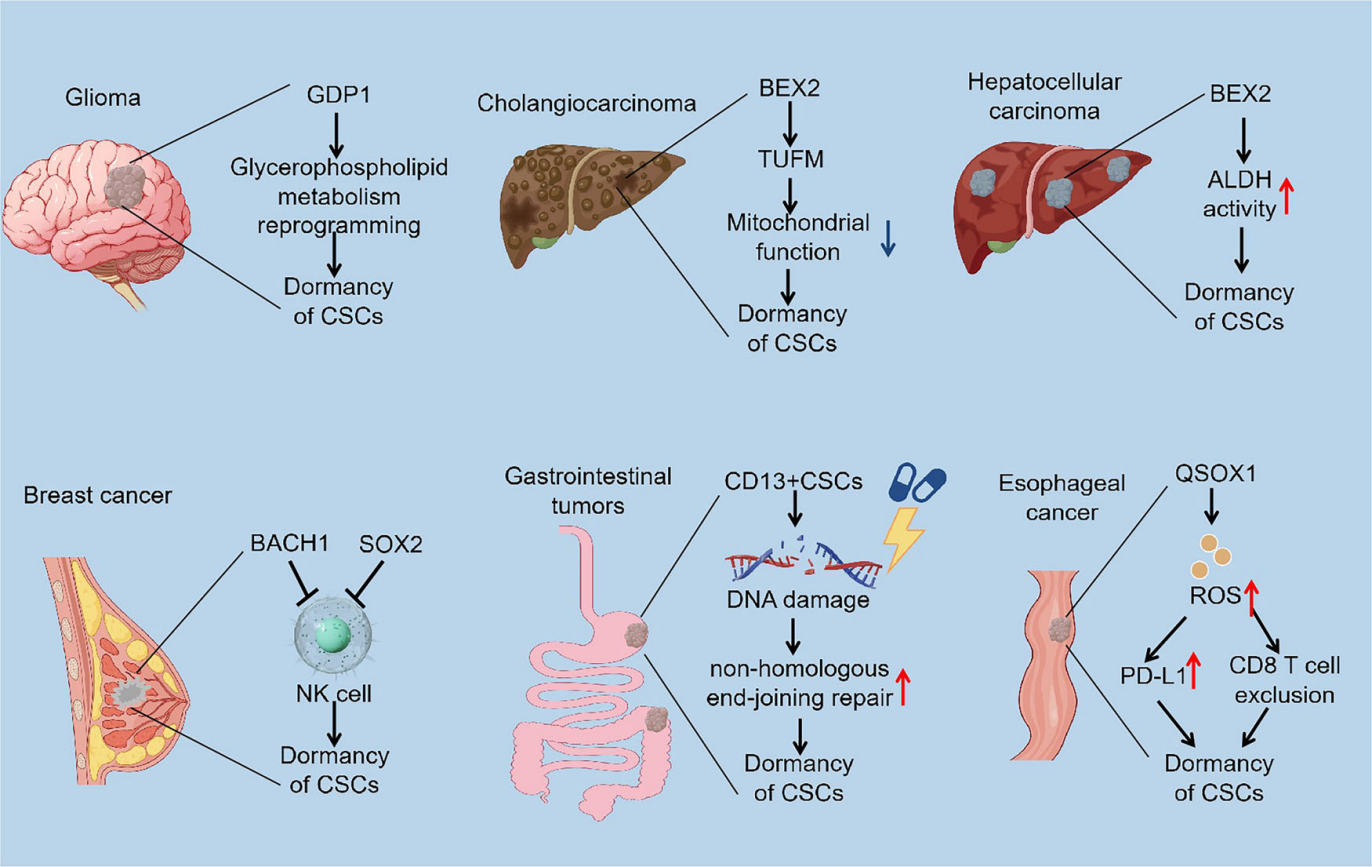
Dormancy characteristics of cancer stem cells from different cancer types. In glioma, dormant tumor stem cells (dormant CSCs) with high expression of glycerol-3-phosphate dehydrogenase 1 (GPD1) have distinct glycerophospholipid metabolism, which promotes the maintenance of CSC dormancy. In cholangiocarcinoma, BEX2 inhibits mitochondrial function by binding to the mitochondrial protein TUFM to maintain the dormancy of cancer stem cells (CSCs). In hepatocellular carcinoma, BEX2 promotes dormancy maintenance in dormant CSCs by increasing the activity of the ALDH enzyme. In breast cancer, dormant CSCs escape NK cell cytotoxicity by upregulating BACH1 and SOX2. In gastrointestinal tumors, CD13(+) dormant CSCs employ an error-prone nonhomologous end-joining repair mechanism to repair DNA damage caused by chemotherapy, which helps maintain the dormant state. In esophageal cancer, QSOX1 promotes dormant CSCs to evade immune elimination through PD-L1 upregulation and CD8 T-cell exclusion.

The dormancy mechanisms of different tumors vary significantly (Fig. 4). In glioma, dormant CSCs with high expression of glycerol-3-phosphate dehydrogenase 1 (GPD1) exhibit distinct glycerophospholipid metabolism. Moreover, these dormant CSC populations are enriched mainly at tumor margins and drive cancer relapse after chemotherapy. The inhibition of GPD1 extended the survival duration of glioblastoma model mice, in part by modulating cellular metabolism and protein translation, which consequently disrupted the maintenance of dormancy in dormant CSCs [57]. In cholangiocarcinoma, BEX2 inhibits mitochondrial function by binding to the mitochondrial protein TUFM to maintain cancer stem cell dormancy [58]. In the context of hepatocellular carcinoma, BEX2 is expressed mainly in Ki67-negative (nonproliferative) cancer cells. BEX2 overexpression significantly increased the quiescent cell population, indicating that BEX2 plays an important role in dormant CSCs. Moreover, BEX2 reduces the chemosensitivity of dormant CSCs to cisplatin by increasing the activity of the ALDH enzyme [59]. In breast cancer, enhanced autophagy promotes the self-renewal capacity of dormant CSCs. Notably, the inhibition of autophagy by either genetic or pharmacological approaches can significantly reduce the risk of dormant breast cancer metastasis [60]. In gastrointestinal tumors, CD13(+) dormant CSCs employ an error-prone nonhomologous end-joining repair mechanism to repair DNA damaged by chemotherapy, whereas CD13(-) proliferating cells utilize high-fidelity homologous recombination to repair DNA damage, indicating that dormancy is an important mechanism of therapeutic resistance [61]. In esophageal cancer, QSOX1 promotes dormant CSCs to evade immune elimination through PD-L1 upregulation and CD8 T-cell exclusion [62]. In glioblastoma, the increase in FOXG1 expression, in synergy with Wnt/β-catenin activation, induces dormant CSCs to exit the quiescent state [63]. In prostate cancer, homeobox B9 promotes the metastasis of dormant prostate cancer stem cells through the TGF-β pathway [64]. Expressed SOX2 can indirectly activate dormant prostate cancer cells through the downstream target gene Cyclin E2 (CCNE2), thereby influencing the progression of cancer and bone metastasis [65]. In head and neck squamous cell carcinoma (HNSCC), tumor-derived type III collagen is crucial for maintaining tumor dormancy because it hinders tumor cell proliferation via DDR1-mediated STAT1 signaling [66]. Furthermore, hypoxia in HNSCC tumors can induce dormant CSCs to enter into the circulation, which is part of the niche defense of platinum therapy [67]. In melanoma, low levels of Sox2 are essential for maintaining the dormancy of cancer stem cells. Interestingly, complete Sox2 knockout results in the termination of dormancy and the subsequent growth resumption of melanoma stem cells in culture [68]. In addition, in patients with melanoma, high expression of IFN-β is associated with tumor cell dormancy. Activation of the STAT3/p53 signaling pathway disrupts IFN-β-induced tumor cell dormancy, resulting in tumor recurrence [69]. In conclusion, the above studies highlight the significant differences in dormancy mechanisms of dormant CSCs in different cancer types.

## Markers of dormant cancer stem cells

The identification and characterization of molecular markers specific to dormant CSCs is essential for the development of precise, targeted tumor therapies [70]. Recently, several new specific markers of DCs, such as p27, CD13, QSOX1, Survivin, GPD1 and BEX2, were identified, suggesting their potential for targeted therapy (Table 2). Cell cycle regulatory factors that keep cells in the G0 phase can be used as markers for dormant CSCs. Several studies have shown that dormant CSC populations have high levels of cyclin-dependent kinase inhibitors (CDKIs), such as p21, p27 and p16. These CDKIs prevent cells from entering S phase from G0 phase by specifically inhibiting the phosphorylation activity of cyclin-dependent kinases (CDKs), thereby maintaining cancer stem cell dormancy [71, 72]. For example, dormant colon cancer stem cells express p27. Therefore, the LGR5 + p27+ cell subpopulation is regarded as dormant CSCs, which can cause cancer relapse after chemotherapy. Mechanistically, chemotherapy disrupts COL17A1 expression and breaks the dormancy of LGR5 + p27+ cells by activating FAK–YAP signaling. Inhibiting YAP signaling can keep LGR5 + p27+ cells in a dormant state and delay cancer relapse, highlighting the therapeutic potential of YAP inhibition in preventing cancer recurrence [72].

**Table 2:** Summary of well-established and potential dormant CSC markers.

Well-established dormant CSC marker	Function(s) in dormant CSCs	The mechanism(s) by which CSCs maintain dormancy	Tumor type(s)	Reference
LGR5 + p27+	p27 promotes cell cycle arrest in G0/G1 phase	Blocking FAK-YAP signaling can prevent dormant CSCs from exiting their dormant state and delay tumor regeneration	Colon cancer	[72]
CD13 + CD90-	CD13 promotes the cell to enter G0 phase, while CD90 promotes cell proliferation	CD13 helps reduce ROS-induced DNA damage and protects cells from apoptosis after radiotherapy and chemotherapy	Hepatocellular carcinoma	[19]
GPD1	GPD1 specifically marks dormant glioma stem cells and maintains their dormancy	GPD1 maintains dormancy by regulating lipid metabolism	Glioma	[57]
BEX2 + Ki67-	Knocking out BEX2 can significantly reduce the self-renewal ability and chemotherapy resistance of CSCs	BEX2 increases aldehyde dehydrogenase (ALDH) activity, thereby helping dormant CSCs evade chemotherapy drug-mediated death	Hepatocellular carcinoma	[59]
NR2F1	NR2F1 promotes the maintenance of dormancy in CSCs	NR2F1-dependent dormancy is epigenetically regulated	HNSCC, melanoma and breast cancer	[75, 79, 80]
**Potential dormant CSC marker**	**Function(s) in dormant CSCs**	**Pathway(s) involving dormant CSCs**	**Tumor type**	**Reference**
QSOX1 (should be combined with stem cell markers such as CD44, CD133, or ALDH1)	QSOX1 helps dormant CSCs evade immune surveillance and maintain their dormant state	PD-L1 overexpression induced by QSOX elicits an immune evasive effect on CD8 + T cells and helps dormant esophageal CSCs evade the immune system	Esophageal cancer	[62]
Survivin (should be combined with stem cell markers such as CD44 or CD133)	Survivin promotes self-renewal, tumor initiation, and the chemoresistance of dormant breast CSCs	SURVIVIN upregulates the WNT/β-CATENIN pathway in a PI3K/AkT-dependent manner to promote the self-renewal of dormant breast CSCs	Breast cancer	[73]

Recent studies have shown that tumor-derived type III collagen in the extracellular matrix (ECM) is required to maintain tumor dormancy, as the degradation of type III collagen restores tumor cell proliferation through DDR1-mediated STAT1 signaling. Moreover, when the type III collagen content in the ECM increased in a mouse model, the dispersed dormant CSCs were forced to enter a dormant state, suggesting that type III collagen in the ECM may serve as a marker of cancer stem cell dormancy [68]. In breast cancer, the survivin protein has been identified as a specific biomarker for quiescent breast cancer stem cells (Q-BCSCs). Mechanistically, survivin increases the activity of the WNT/β-CATENIN pathway in a PI3K/AKT-dependent manner to promote the dormancy of Q-BCSCs [73]. In esophageal cancer, quiescin sulfhydryl oxidase 1 (QSOX1), which is secreted by tumor-associated fibroblasts, may serve as a marker of cancer stem cell dormancy. QSOX1 can significantly upregulate PD-L1 in dormant CSCs, thereby helping them evade immune surveillance and maintain their dormant state [62].

Recent research has revealed that GPD1 is highly expressed in dormant glioma stem cells and maintains their dormancy by regulating glycerophospholipid metabolism. The inhibition of GPD1 can significantly prolong the survival of animal models, and drugs targeting GPD1 are currently in preclinical research [57]. BEX2 has been identified as a biomarker for dormant liver cancer stem cells in the context of hepatocellular carcinoma, and its expression level is closely related to patient prognosis. According to immunohistochemical analyses, BEX2 localizes to Ki67-negative (nonproliferative) tumor cell subsets. Functional studies have shown that knocking out BEX2 can significantly reduce the self-renewal ability and chemotherapy resistance of dormant CSCs [59]. Previous studies have shown that CD13 is a biomarker of dormant liver CSCs [19]. CD13 helps reduce ROS-induced DNA damage and protects cells from apoptosis after chemoradiotherapy[19].

A population of quiescent cancer stem cells with a CD13 + CD90- phenotype was found in gastrointestinal tumors. These cells are characterized by limited proliferation and survival in hypoxic environments and are associated with cancer relapse and metastasis. Notably, CD13 + CD90- dormant CSCs primarily use an error-prone nonhomologous end joining mechanism to repair DNA damage caused by chemotherapy, whereas CD13-CD90+ proliferative cells favor a more accurate homologous recombination repair pathway. This difference in repair mechanisms may drive their distinct biological behaviors [61]. A recent study identified a stem cell-like subpopulation (CD24-CD44 + ESA+ phenotype) upon the stimulation of MDA-MB-231 cells via hypoxia/reoxygenation stress, potentially offering a new molecular marker for the clinical detection of dormant breast cancer stem cells [22, 27]. NR2F1-dependent dormancy is epigenetically regulated in various types of cancer [74–77]. In an experimental head and neck squamous cell carcinoma model, the restoration of NR2F1 expression using the DNA demethylating agents 5-Aza-C and retinoic acid can induce a dormant phenotype that persists for at least 2 weeks [78]. In the initial phases of breast cancer progression, the suppression of NR2F1 expression mediated by HER2 facilitates dissemination by inducing epithelial‒mesenchymal transition and promoting a hybrid luminal/basal-like phenotype [79]. Recently, Tiago et al. reported the role of the dormancy marker NR2F1 as a key determinant of therapeutic resistance in BRAF-mutant melanoma [80]. The over-expression of NR2F1 was adequate to attenuate the effects of BRAF-V600E inhibitors (BRAFi) plus MEK inhibitors (MEKi) on tumor growth in vivo, as well as on cell proliferation and invasion in vitro. The findings suggest that targeting NR2F1 with mTORC1 inhibitors could potentially enhance therapeutic outcomes in melanoma patients [80]. On the other hand, the use of NR2F1 agonists to induce dormancy in HNSCC cells may serve as a therapeutic strategy for preventing metastasis [75]. Therefore, different targeting strategies are employed for NR2F1. Agonists can be used to maintain the tumor’s dormant state, while inhibitors can restore the proliferative state of tumor cells, allowing them to be subsequently killed by chemotherapeutic drugs.

## Challenges and limitations in identifying markers of dormant CSCs

Although we have described several promising dormant CSC markers (such as p27, CD13, QSOX1 and BEX2) in the previous text, these markers are not unique to dormant CSCs. Their specificity is indeed a key challenge, as these markers can also be expressed in dormant cancer cells with non-stem cell characteristics and even dormant cells in normal tissues (such as stem cells, hematopoietic stem cells, and quiescent lymphocytes) [81–87]. Therefore, critically evaluating the limitations of these markers is crucial for advancing reliable research in this field.

P27 is a universal biomarker of cell cycle arrest [88]. Any cell in the G0 phase, whether it is normal or cancerous or a stem cell or non-stem cell, may have increased levels of p27 [89, 90]. Therefore, p27 is more suitable for indicating the dormant state of cells rather than their stem cell characteristics. p27 must be used in combination with other true stem cell markers (such as Lgr5 + , CD44+ or CD133 + ) to identify dormant CSCs.

CD13 is highly expressed in chemotherapy-induced dormant liver cancer cells [91]. However, CD13 is also widely expressed in various normal cells, such as myeloid cells, endothelial cells, and epithelial cells, and tumor-associated macrophages [92–95]. The specificity of CD13 is highly dependent on the tumor type and microenvironment. There may be a strong correlation between dormant CSCs and CD13 expression in liver cancer lesions, but caution should be exercised when examining other types of cancer tissue. It is thus necessary to combine epithelial markers, such as EpCAM, to exclude CD13+ cells from non-epithelial sources.

QSOX1 is a secreted enzyme [96]. In a model of breast cancer dormancy, its high expression is related to dormancy, and dormancy may be maintained by remodeling the extracellular matrix [97, 98]. As QSOX1 is a secreted protein, its detection (such as in serum or tissue fluid) may reflect the overall characteristics of the microenvironment rather than specific membrane surface markers of dormant CSCs. It is difficult to determine whether high levels of QSOX1 are derived from a small number of dormant CSCs or a large number of proliferating tumor cells or stromal cells [99–101]. QSOX1 may be more suitable as an indirect indicator of or potential therapeutic target for microenvironmental dormancy than for the direct isolation and identification of dormant CSCs.

In summary, molecular markers such as p27, CD13, and QSOX1 provide important entry points for the study of dormant CSCs, but they lack specificity and reliability as independent markers. Given the limitations of individual markers, future research should use a multiparameter marker combination strategy to improve the reliability of identifying dormant CSCs, namely, “dormant markers (such as p27)”+“stem cell markers (such as Lgr5, CD44, CD133, and ALDH1)”+“tumor-specific markers (such as epidermal cell adhesion molecule (EpCAM) or tumor-specific antigens). For example, compared with p27 alone, p27 + /Lgr5+ cells are more representative of dormant CSCs [72, 102].

## The molecular mechanism underlying the dormant state of dormant CSCs

### Communication between dormant CSCs and the tumor microenvironment

**Fig. 5:**
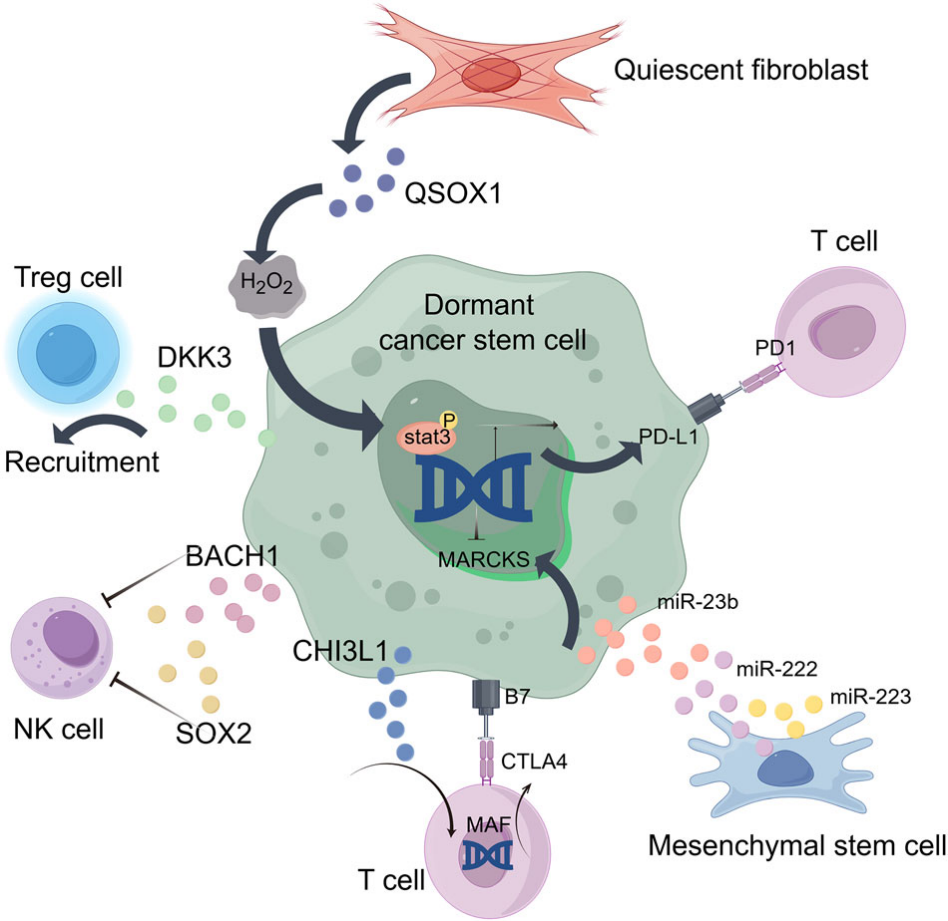
Influence of the tumor immune microenvironment on dormant cancer stem cells. QSOX1, which is secreted by tumor-associated fibroblasts within the microenvironment, promotes the production of reactive oxygen species and induces PD-L1 expression in dormant esophageal cancer stem cells. PD-L1 overexpression induced by QSOX1 elicits an immune effect on CD8 + T cells and helps dormant esophageal cancer stem cells evade the immune system. Dormant breast cancer stem cells can secrete the DKK3 protein, which creates an immunosuppressive microenvironment enriched with regulatory T cells (Tregs) to ensure immune evasion. Dormant breast CSCs escape NK cell cytotoxicity by upregulating BACH1 and SOX2. In triple-negative breast cancer, CHI3L1 secreted by cancer stem cells mediates immune escape by activating the MAF/CTLA4 pathway. Bone marrow-derived mesenchymal stem cells (MSCs), via their secretion of exosomes enriched with miR-23b, can suppress MARCKS expression in metastatic breast cancer cells, which results in their dormancy. Moreover, MSCs can cause tumor cell dormancy and create drug-resistant phenotypes through the effects of exosomes containing miR-222/223.

Dormant CSCs closely communicate with various immune cells within the tumor microenvironment (Fig. 5). Approximately 30% of breast cancer patients may experience local and/or distant relapses many years after their first diagnosis because of a phenomenon known as metastatic dormancy [103, 104]. Studies have shown that NK cells play dual roles in dormancy maintenance and immune surveillance [105]. Metastatic dormancy is a dynamic process in which tumor cells can switch between dormant and proliferative states. Dormant CSCs are resistant to NK cell cytotoxicity, possibly because of their low expression of MHC-I and quiescent metabolic state [106–108]. Moreover, Bushnell et al. revealed that dormant breast CSCs escape NK cell cytotoxicity by upregulating BACH1 and SOX2 [43]. However, proliferating tumor cells are sensitive to NK cell cytotoxicity [109, 110]. The role of NK cells in metastatic dormancy lies in their immune surveillance function, which effectively identifies and eliminates tumor cells that attempt to exit dormancy and resume proliferation [107]. In addition, cytokines secreted by NK cells, such as IFN-γ, may create an inhibitory “dormant niche” in the microenvironment by inhibiting angiogenesis and inducing direct antiproliferative effects [111]. In conclusion, NK cells regulate metastatic dormancy by controlling proliferating tumor cell populations, not by uniformly killing dormant cells. In addition, tumor-associated macrophages can also activate the dormancy program in disseminated tumor cells [112]. The depletion of macrophages leads to a reduction in dormant cells among primary tumors, circulating tumor cells, and disseminated tumor cells in vivo [113].

Hypoxia serves as a microenvironmental hallmark of an unfavorable prognosis in solid tumors [114]. Research has demonstrated that hypoxia substantially influences the fate of disseminated tumor cells within target organs [115, 116]. For instance, dormancy-related genes (NR2F1, DEC2, and p27) are significantly upregulated in the hypoxic microenvironments of head and neck squamous cell carcinoma (HNSCC) and breast cancer [117]. Furthermore, low-oxygen environments give rise to a subpopulation of dormant disseminated tumor cells that cause cancer relapse [117].

In esophageal cancer tissues, dormant CSCs closely communicate with resting fibroblasts to help them evade immune surveillance [118]. Studies have shown that QSOX1, which is secreted by resting fibroblasts within the microenvironment, promotes dormant esophageal cancer stem cells to evade immune elimination via PD-L1 upregulation and CD8 T-cell exclusion [62]. Clinically, high expression levels of QSOX1 may serve as a key biomarker to predict poor response to anti-PD-1 therapy in esophageal cancer patients [62]. Blocking QSOX1 with ebselen in combination with anti-PD-1 therapy and chemotherapy can effectively eliminate residual esophageal cancer stem cells, representing a promising treatment method for eliminating DCSCs and preventing recurrence [62].

In breast cancer, chemotherapy can induce lung fibroblasts to enter a senescent state and stimulate neutrophils to form extracellular traps (NETs) through the release of senescence-related secretory factors. Furthermore, NETs promote the proliferation of dormant CSCs by reshaping the extracellular matrix, eventually leading to lung cancer metastasis. Therefore, eliminating senescent fibroblasts in the lung cancer microenvironment after chemotherapy may help dormant CSCs maintain dormancy and prevent lung metastasis after chemotherapy [119]. In addition, dormant breast cancer stem cells can secrete the DKK3 protein, which creates an immunosuppressive microenvironment enriched with regulatory T cells (Tregs) to ensure immune evasion [120].

Notably, bone marrow-derived mesenchymal stem cells (MSCs) play a crucial role in the tumor dormancy regulatory network through exosome-mediated intercellular communication. For instance, bone marrow-derived MSCs can inhibit the expression of MARCKS in breast cancer cells by secreting exosomes rich in miR-23b, leading to tumor cell dormancy [121]. Furthermore, according to another study, bone marrow-derived MSCs can cause tumor cell dormancy and generate drug-resistant phenotypes through exosomes containing miR-222/223 [122]. Marrow-niche-derived GAS6 is regulated by Mer/mTOR, which is crucial for maintaining the dormant state of prostate cancer stem cells[123]. Studies have shown that non-proliferative disseminated tumor cells (DTCs) can persist in the bone marrow, while other organs (such as the lungs) develop growing metastases. This suggests that the bone marrow may serve as a “restrictive microenvironment” for DTCs, whereas the lungs may function as a “permissive soil” for DTCs. In the bone marrow, specific transforming growth factor-β2 (TGF-β2) signaling activates the MAPK p38α/β pathway, which induces DEC2/SHARP1 and p27 to promote dormancy in DTCs. In contrast, in the lungs—a “fertile soil” for metastatic tumors—where TGF-β2 levels are low, DTCs undergo a brief dormant period before subsequently initiating metastatic growth. Another study revealed differences between the lung and brain metastatic microenvironments: lung-derived BMPs promote dormancy maintenance in DTCs, whereas the bone and brain microenvironments lack bioactive BMP. Therefore, the BMP inhibitor Coco reactivates DTCs at lung metastatic sites but fails to activate DTCs in bones and brains. In summary, there is high heterogeneity in the microenvironment among different tumor types, between primary and metastatic tumors, and even across different metastatic sites. This results in the diversity and complexity of dormancy regulatory signals. Therefore, a comprehensive understanding of tumor dormancy should consider the specific microenvironmental context. Further studies are needed to elucidate how these signals vary across different metastatic niches.

### Role of signaling pathways in cancer stem cell dormancy

**Fig. 6:**
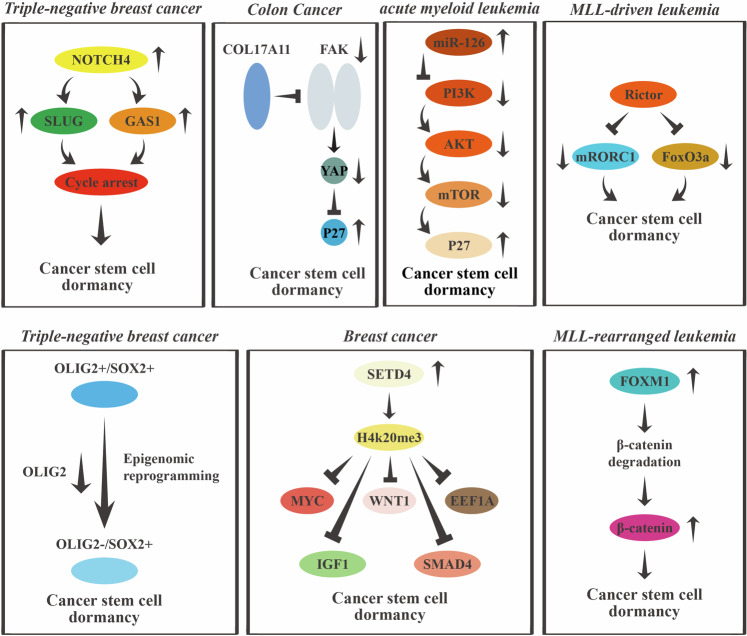
Roles of signaling pathways in cancer stem cell dormancy. In triple-negative breast cancer, the NOTCH4 signaling pathway maintains the dormancy of breast cancer stem cells with mesenchymal characteristics state by upregulating two key genes, SLUG and GAS1. In colon cancer, chemotherapy-induced activation of the FAK–YAP pathway promotes the degradation of COL17A1 and the reactivation of LGR5 + p27+ dormant cancer stem cells. miR-126 maintains the dormant phenotype and increases the chemotherapy resistance of human acute myeloid leukemia stem cells via the PI3K/AKT/mTOR pathway. The Rictor protein, a component of the mTORC2 complex, may play a role in maintaining the dormancy of leukemia stem cells through the FoxO3a signaling pathway. In medulloblastoma, OLIG2 drives SOX2+ cancer stem cells to switch from quiescence to active proliferation, and OLIG2 inhibition induces tumor regression. The SETD4 protein catalyzes the histone modification H4K20me3 at the epigenetic level, which promotes heterochromatin formation and regulates the dormant state of breast cancer stem cells. The transcription factor Foxm1 plays a crucial role in Wnt signaling activation by stabilizing β-catenin and maintains the quiescence of leukemia stem cells and enhances their self-renewal capacity.

Many signaling pathways collectively control the quiescent state of cancer stem cells (Fig. 6). mTOR is an important regulatory player that affects the quiescent state of cancer stem cells through the regulation of cell growth and metabolism. Under conditions of nutritional deficiency or hypoxia, blocking the mTOR pathway prompts head and neck squamous cell carcinoma stem cells to enter a dormant state with reduced metabolism [124]. In contrast, the activation of the mTOR pathway has the opposite effect [125]. In the context of acute myeloid leukemia, miR-126 maintains the dormant phenotype of dormant CSCs and increases their resistance to chemotherapy via the PI3K/AKT/mTOR pathway [126]. The Rictor protein, a component of the mTORC2 complex, also plays a key role in maintaining the dormancy of leukemia stem cells through the FoxO3a signaling pathway [127]. In addition to the mTOR pathway, the Notch, SETD4, Hedgehog and Wnt/β-catenin pathways are involved in regulating the dormancy of cancer stem cells and are potential therapeutic targets [128, 129]. For instance, in triple-negative breast cancer cells, the NOTCH4 signaling pathway upregulates two key genes, SLUG and GAS1, inducing breast cancer stem cells with mesenchymal characteristics to enter a dormant state, thereby enhancing their metastatic ability and drug resistance [130]. The SETD4 protein catalyzes histone H4K20me3 modification to promote the formation of heterochromatin and regulate the dormant state of breast cancer stem cells. In dormant breast cancer stem cells, H4K20me3 modification is specifically enriched in promoter regions and regulates the expression of genes such as MYC, WNT1, EEF1A1, IGF1 and SMAD4, which causes chemoresistance and tumor recurrence [131]. In medulloblastoma cells, OLIG2 drives the transformation of SOX2+ cancer stem cells from a quiescent state to a proliferative state, whereas OLIG2 inhibition induces tumor regression [132]. Furthermore, the Wnt signaling pathway plays a crucial role in maintaining the dormancy of leukemia stem cells [133]. Foxm1 promotes the quiescent state of leukemia stem cells by stabilizing β-catenin [134]. In glioblastoma, FOXG1 functions together with Wnt signaling to promote the transition of cancer stem cells from dormancy to proliferation [135]. Previous studies have shown that high expression levels of YAP are closely associated with colon cancer relapse [136]. In the context of colon cancer, FAK–YAP signaling regulates the balance between the dormancy and proliferation of cancer stem cells [72]. Moreover, blocking the FAK–YAP pathway can sustain cancer stem cell dormancy and delay tumor recurrence [72]. The activation of the FAK–YAP pathway caused by chemotherapy promotes the degradation of COL17A1 and the awakening of LGR5 + p27+ dormant colorectal CSCs.

### Dynamic interactions between chemotherapy and cancer stem cells: awakening, selection and induction

**Fig. 7:**
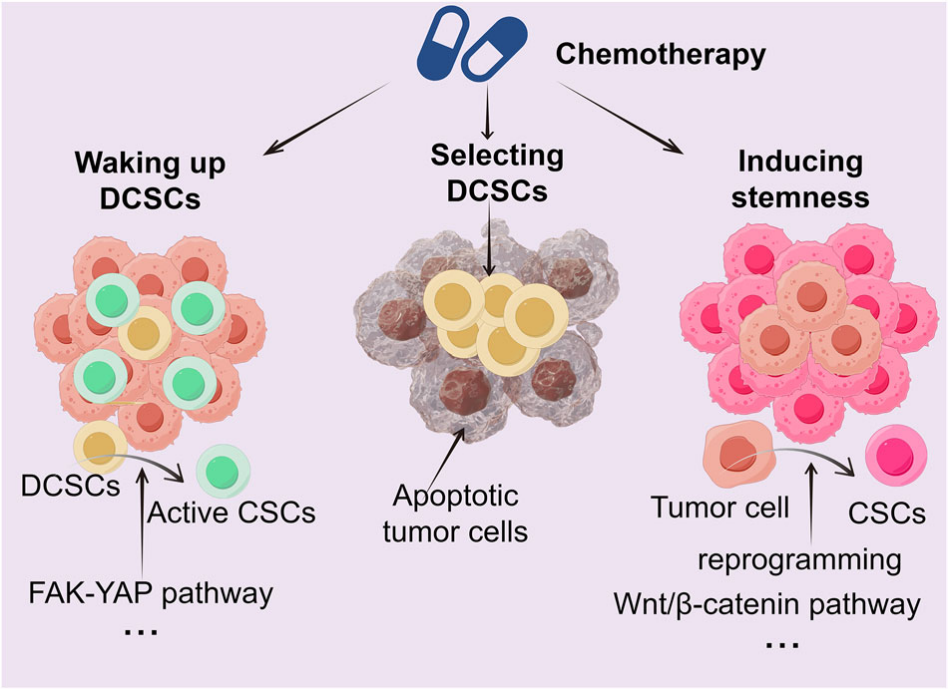
Multiple effects of chemotherapy on cancer stem cells (CSCs). Chemotherapy not only awakens dormant CSCs but also selects for dormant CSCs (DCSCs) or induces stemness.

As mentioned above, chemotherapy can indeed awaken dormant CSCs, potentially causing therapeutic resistance and relapse [72]. Notably, chemotherapy can also select for dormant cells or induce stemness (Fig. 7). Chemotherapy, as a powerful selective pressure, preferentially kills tumor cells that are actively proliferating (mostly non-CSCs) [137]. However, dormant CSCs can survive under such pressure because of their inherent characteristics, such as cell cycle arrest, high DNA repair capacity, and increased expression of the ABCG2 efflux pump [38, 136, 138–141]. Therefore, the relative proportion of dormant CSCs in the tumor microenvironment may increase after treatment. In addition, chemotherapy can also endow tumor cells with stemness characteristics. Tumor plasticity refers to the ability of tumor cells to transition between different states [142]. DNA damage, oxidative stress or microenvironmental changes caused by chemotherapy can activate a series of stemness-related signaling pathways. These pathways reprogram tumor cells, endowing them with stem cell-like characteristics such as self-renewal and multidirectional differentiation potential [143–145]. For example, cisplatin can increase the expression of stemness markers such as CD44 and CD133 in lung cancer tumor cells, indicating that chemotherapy can induce stemness [146]. In conclusion, these findings reflect the multiple effects of chemotherapy on tumor stem cells. Chemotherapy not only awakens dormant CSCs but also selects for dormant cells or induces stemness, potentially causing therapeutic resistance and relapse.

### Role of epigenetic modifications in cancer stem cell dormancy

The areas of the genome that are rich in CpG dinucleotides are sites of methylation that are critical for gene expression regulation [147]. DNA methylation is important for the regulation of tumor stem cell dormancy [148]. For instance, in oral cancer cells, the demethylation of the Sox2 promoter promotes its gene expression, thereby driving cancer stem cells from the quiescent phase to a proliferative state [149]. Dormant CSCs derived from glioblastoma tissue have specific DNA methylation profiles, which are characterized by the downregulation of some tumor suppressor genes, such as SPINT2, NEFM and PENK [150]. Notably, Cdc42 is a mechanical signal transduction protein that can sense changes in matrix stiffness and translocate to the nucleus, subsequently upregulating Tet2 hydroxymethyltransferase. Tet2 maintains the dormancy of cancer stem cells by mediating the epigenetic activation of cyclin-dependent kinase inhibitors such as p21 and p27 [71]. In addition to DNA methylation, histone modification also regulates the transition between the quiescent and proliferative states of CSCs. Notably, lactate can regulate tumor dynamics by inhibiting the differentiation of CSCs and inducing their dedifferentiation into a proliferative state. Mechanistically, lactate can promote histone acetylation, thereby epigenetically activating MYC [151]. These findings indicate that lactate may promote the end of dormancy and maintain the proliferative state of dormant CSCs through the epigenetic activation of MYC. The acetyltransferase KAT5, another epigenetic regulator, also plays a key role in the state transition of glioblastoma stem cells. KAT5 knockout promotes glioma stem cells to enter a dormant state and effectively inhibits tumor growth and invasion ability [152]. In addition, the shortest isoform of the histone demethylase LSD1 can drive quiescent hematopoietic stem cells (HSCs) to form preleukemic HSCs with increased self-renewal potential [153]. Ferrer-Diaz and colleagues reported that the H3K4 methyltransferases KMT2B and KMT2D increase the chemoresistance of breast CSCs by maintaining their dormancy. In contrast, knocking out these two enzymes can awaken dormant breast CSCs [154]. Furthermore, Fujita et al. reported that PRC2 maintains the stemness of leukemia stem cells by catalyzing the trimethylation of histone H3K27. Knocking out the catalytic subunit EZH1/2 of PRC2 can effectively eliminate dormant leukemia stem cells and alleviate AML [155]. By targeting γ-catenin, Jin et al. confirmed that histone deacetylase inhibitors can effectively eliminate dormant tumor stem cells in chronic myeloid leukemia [155]. In summary, epigenetic regulators may serve as potential targets for regulating cancer stem cell dormancy.

## Models used for studying dormancy

**Fig. 8:**
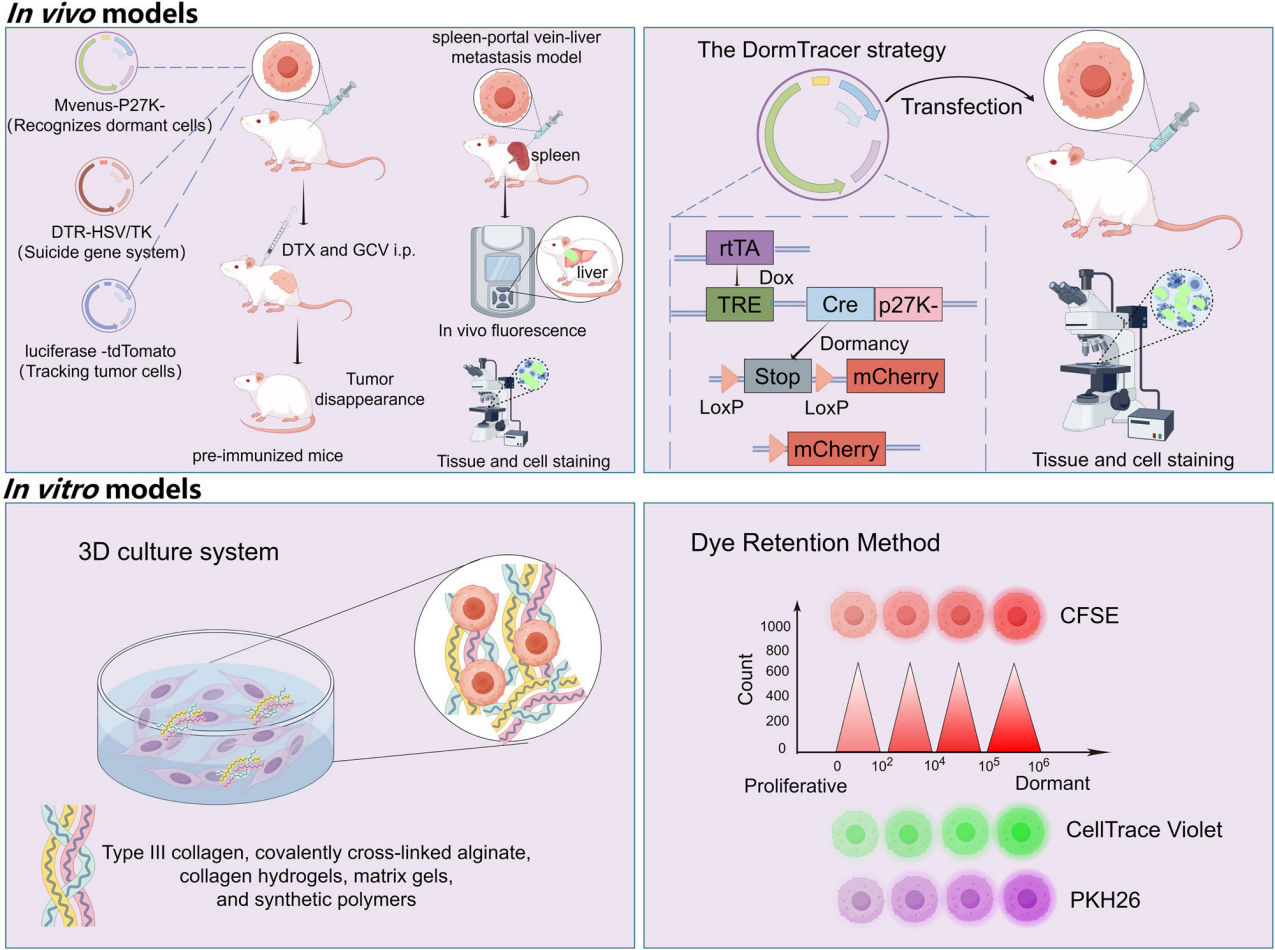
In vivo and in vitro models for studying tumor dormancy. Preimmunization models and the DormTrace reporting system allow for the visualization and tracking of dormant cancer stem cells in vivo on the basis of cell cycle status. 3D culture systems mimic dormancy-inducing environmental cues such as low oxygen levels and extracellular matrix structure. Dye retention-based methods allow for the visualization and tracking of dormant CSCs on the basis of dye dilution. CFSE, carboxyfluorescein succinimidyl ester.

The use of in vitro and in vivo models for studying dormancy is very important for translational research (Fig. 8). Preimmunization in mice involves the subcutaneous injection of tumor cells to enable the mouse’s immune system to recognize tumor-associated antigens and form memory T and B cells [156]. When metastatic tumor cells subsequently enter the liver, the preexisting immune cells can quickly recognize and inhibit their proliferation, but also allow them to enter the G0 phase dormant state [157–160]. By integrating the preimmunization strategy with the mVenus-p27K- cell G0 phase indicator system, the DTR-HSV/TK suicide gene system [161, 162] and the luciferase-tdTomato reporter [163], a tumor-dormant mouse model without obvious metastasis was successfully constructed [160, 164–166]. First, the cancer cell line was engineered to coexpress the luciferase-tdTomato reporter and the fluorescent fusion protein mVenus with the mutant form of the cell cycle inhibitor p27 (Mvenus-P27K-), which recognizes dormant cells [157]. Then, these cells were engineered to express DTR-HSV/TK. Interestingly, cells transfected with the DTR system express the diphtheria toxin receptor, which binds to diphtheria toxin and promotes apoptosis. Moreover, cells transfected with the HSV/TK system express thymidine kinase of herpes simplex virus, which can convert nontoxic ganciclovir (GCV) into lethal metabolites and cause cell death. The preimmunization model, combined with the multifunctional reporting system, is constructed as follows. Tumor cells subcutaneously injected into C57BL/6 mice can activate the mouse immune system, enabling them to recognize and acquire the ability to kill tumor cells. DTX and GCV are subsequently used to induce tumor cell death, resulting in preimmunized mice. A spleen-portal vein-liver metastasis model was constructed using preimmunized mice. After splenectomy, only a small number of tumor cells remained in the mice. The mVenus-p27K- system was used to determine the cell cycle, confirming that the tumor cells in the mice had entered G0 phase [167]. Therefore, this model is ideal for studying tumor dormancy.

Another system used for tracking dormant cells in vivo is DormTracer, which consists of a doxycycline-inducible p27K fused with Cre recombinase, with an mCherry locus located after the LoxP-flanking stop element. Doxycycline can trigger Cre-p27K- expression; however, its recombinase function is inhibited in proliferating cells because p27 is degraded in the cytoplasm. When a cell becomes dormant, it removes the stop element, permanently activating mCherry. Thus, in the presence of doxycycline, DormTracer continuously tracks cells entering dormancy. He et al. used the recombinase-based DormTracer system to confirm that the reactivation of chemotherapy-induced dormant disseminated tumor cells can cause metastasis and recurrence [168].

Two-dimensional (2D) culture systems are fundamental in cancer cell research, but replicating the complexity of in vivo conditions with these systems is difficult, making it challenging to translate in vitro findings to clinical settings. To overcome these limitations, a 3D culture system was created to more accurately replicate the physiological and mechanical traits of the in vivo microenvironment. Researchers have used a 3D culture system to mimic the structure and mechanics of the extracellular matrix (ECM), offering a more authentic model for studying tumor dormancy. Type III collagen, covalently cross-linked alginate, collagen hydrogels, matrix gels, and synthetic polymers are typically used to recreate dense ECM conditions that trigger tumor dormancy [66, 169, 170].

The dye retention method is a unique approach for studying dormant cells in vitro. This method uses fluorescent dyes, such as carboxyfluorescein succinimidyl ester (CFSE), PKH26 (a lipophilic dye) and CellTrace Violet (a cell-permeable dye), to label cells on the basis of their division rate [171]. Researchers can differentiate between rapidly dividing and dormant cells, as the fluorescence signal weakens with each cell division. CFSE is typically used for tracking dormant cell populations in vitro. With each cell division, the concentration of CFSE is diluted approximately twofold, enabling dormant cells to retain greater fluorescence intensity [172]. This offers a simple and cost-effective way to distinguish dormant cells. However, this method has certain limitations, such as fluorescence fading, which restricts its long-term application.

Hypoxia, a hallmark of the tumor microenvironment (TME), plays a pivotal role in the induction and maintenance of tumor dormancy [114]. CSCs that disseminate to distant organs often reside in low-oxygen, poorly vascularized niches, a phenomenon known as “angiogenic dormancy”. A study conducted by Fluegen et al. revealed that under hypoxic conditions, the expression of dormant markers, including TGF-β, NR2F1, and p27, is increased in tumors, thereby facilitating the dormancy of disseminated tumor cells [117]. A hypoxic culture system provides a controlled environment for the study of genes and proteins linked to tumor dormancy under low-oxygen conditions. For example, cobalt chloride is used to stabilize hypoxia-induced cellular responses, enabling researchers to study cellular changes via sustained HIF1α expression [173]. However, this method has certain limitations. First, the transient conditions might not accurately mimic chronic hypoxia. Second, this approach requires specialized equipment and precise regulation of the oxygen concentration, making it labor- intensive and technically complex (Table 3).

**Table 3:** In vitro and in vivo models are used for studying dormancy.

Model category	Model name	Core features	Applications
In vivo			
	The preimmunization model combined with the multifunctional reporting system	By using transgenic technology, cells can express specific fluorescent proteins (such as mVenus) during dormancy, thereby directly identifying, tracing and targeting dormant cells.	Simulate dormancy without obvious metastatic foci. Study the in vivo dynamic behaviors of dormant cells [167].
	Splenic-portal vein-liver metastasis model	Tumor cells are injected through the spleen to reach the liver via portal vein circulation, forming micrometastases or dormant cell clusters.	Study the dormancy and activation mechanisms of tumor cells in specific organs, such as the liver [194].
	Immunocompetent models	Using mice with a complete immune system can more realistically simulate the interactions between tumors and the immune microenvironment.	Study the role of immune cells (such as NK cells) in regulating the dormancy and activation of cancer stem cells [43].
	Recombinase-based dormancy tracing system, DormTracer	DormTracer consists of doxycycline-inducible p27K- fused with Cre recombinase, as well as an mCherry locus located after the LoxP-flanking stop element.	DormTracer continuously tracks cells entering dormancy [168].
In vitro			
	2D clonogenic model	Cells are inoculated at a low density on culture plates coated with a matrix such as fibronectin, and specific factors (such as FGF-2) are used to induce the cells to enter a dormant state.	Study the molecular mechanisms of dormancy (such as cell cycle arrest and survival signaling pathways). Conduct preliminary drug screens [195].
	Dormancy-inducing 3D engineered matrix model	The three-dimensional structure formed by a substrate such as alginate gel with increased rigidity can better simulate the physical and chemical properties of the tumor microenvironment in vivo.	This model is helpful for drug screens and seeking therapeutic strategies that can effectively target dormant cells [169].
	Serum deprivation method	By removing essential growth factors such as serum, tumor cells are forced into a dormant state of cell cycle arrest.	Rapidly establish a cell dormancy model. Study the fundamental molecular events related to dormancy [196].
	Dye retention-based models	This method uses fluorescent dyes to label cells according to their division rate.	Detect dormant cells via fluorescence dilution [172].
	Hypoxic culture	This method mimics the oxygen-deprived TME that promotes dormancy.	Induce dormancy-related markers and pathways [173].
Special research systems			
	Model organism-nematode model	Constructing tumor like lesions using *Caenorhabditis elegans* and utilizing its genetic tool advantages for large-scale gene screening.	High-throughput screening of genes related to tumor dormancy [197].
	Osteoclast coculture model	Dormant tumor cells are cocultured with osteoclasts (an important component of the tumor microenvironment) to study their interactions.	To study the effects of activation and mechanism of the bone microenvironment (a common site of tumor metastasis) on dormant tumor cells [198].

## Therapeutic strategies for dormant CSCs

### The development of drugs targeting dormant CSCs

**Fig. 9:**
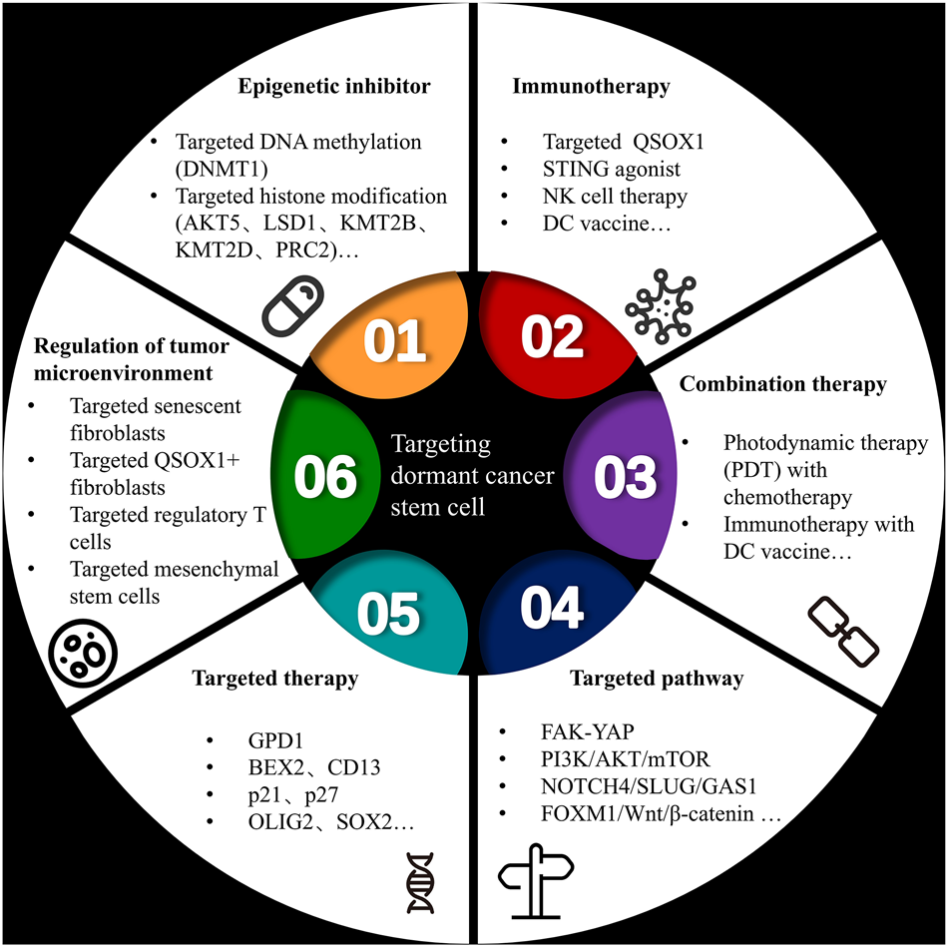
Therapeutic strategies for dormant cancer stem cells. Therapeutic strategies for dormant cancer stem cells include mainly epigenetic inhibitors, immunotherapy, molecular biomarker targeted therapy, pathway targeted therapy and combination therapy.

The development of targeted therapies for dormant CSCs has become an important breakthrough in the field of tumor treatment (Fig. 9). Recent studies have provided evidence that GPD1 regulates glioma stem cell dormancy. GPD1 inhibitors can alter glucose and lipid metabolism and inhibit protein translation in and reduce the self-renewal ability of dormant glioma stem cells. Moreover, GPD1 inhibitors can help extend the lifespan of glioblastoma model mice, positioning GPD1 as a new target for the treatment of brain tumors [57]. In hepatocellular carcinoma tissue, CD13⁺ dormant CSCs are among the main causes of chemotherapy resistance and tumor relapse. Bestatin, a CD13 inhibitor, exhibits antitumor activity when used in combination with low-dose cyclophosphamide [91]. In colon cancer treatment, chemotherapy can activate the FAK–YAP pathway, thereby causing LGR5 + p27+ dormant CSCs to resume proliferation and leading to tumor recurrence. Targeting YAP signaling can delay tumor recurrence [72]. In the context of head and neck squamous cell carcinoma, targeting the RGS2-mediated translational regulatory pathway selectively induces the apoptosis of dormant CSCs [125]. In addition, myosin light chain kinase inhibitors can prevent dormant cancer cells from proliferating. Therefore, regulating the cytoskeleton may become a new target for preventing the activation of tumor cells [174]. The iron-chelating agent Deferasirox reduces the stemness characteristics of tumor cells by interfering with iron metabolism [175]. Furthermore, studies have shown that diphenylhydrazine can selectively induce the apoptosis of dormant CSCs by inhibiting the respiratory chain [176]. Notably, the efficacy of some drugs is enhanced through a “awakening–kill” mechanism. For instance, drugs such as interferon-α, granulocyte colony-stimulating factor (G-CSF), and arsenic trioxide (As₂O₃) can activate dormant leukemia stem cells and promote their proliferation. The application of combined chemotherapy can effectively eliminate cancer stem cells in the proliferative stage, providing a new approach to overcome the drug resistance of cancer stem cells [177].

### Application of immunotherapy in targeting dormant CSCs

Recent studies have shown that immunotherapy strategies targeting dormant CSCs have clinical prospects. QSOX1 promotes immune escape of dormant CSCs by upregulating PD-L1 and excluding CD8⁺ T-cell infiltration. The combination of QSOX1 inhibition, anti-PD-1 treatment and chemotherapy can effectively eradicate residual dormant CSCs. In addition, high QSOX1 expression levels are associated with a low response rate to anti-PD-1 treatment, indicating that QSOX1 can serve as a predictive biomarker for immunotherapy [62]. NK cells play an essential role in breast cancer metastatic dormancy. Dormant breast cancer stem cells exhibit intrinsic resistance to NK cell-mediated cytotoxicity. MSA-2, a STING agonist, can increase the clearance rate of dormant CSCs by NK cells. These findings indicate that it is feasible to develop NK cell-based immunotherapy strategies to eliminate dormant CSCs [43].

Dendritic cells (DCs) are specialized antigen-presenting cells that can trigger specific T-cell-mediated immune responses. Dendritic cells treated with CD44+ colon cancer stem cell lysates induce a strong antitumor immune response, indicating that DC-based vaccines have the potential to be effective against dormant CSCs [178]. In addition, the combination of mechanical high-intensity focused ultrasound (M-HIFU) and a DC vaccine against dormant CSC markers could produce a synergistic antitumor effect. M-HIFU mediates immunogenic tumor cell death, thereby leading to the release of large amounts of tumor-associated antigens and damage-associated molecular patterns (DAMPs), establishing a favorable microenvironment for the immune response. DC vaccines loaded with dormant CSC markers show an improved ability to take up and present tumor antigens, thereby promoting the activation of antigen-specific T cells and facilitating the infiltration of various immune cells into the tumor microenvironment. This approach can prevent the progression of various tumors and provide new information on how to overcome the treatment resistance of dormant CSCs [179]. In the context of breast cancer, dormant CSCs escape NK cell cytotoxicity by upregulating BACH1 and SOX2. Notably, the STING agonist MSA-2 can inhibit the chemotherapy resistance of dormant CSCs and enhance the specific clearance of dormant CSCs by NK cells, providing a potential immunotherapeutic strategy to target dormant CSCs and prevent tumor recurrence [43].

### The application of epigenetic modulators in targeting dormant CSCs

Epigenetic modulators offer new therapeutic approaches to combat dormant CSCs. In the context of breast cancer, targeting the H3K4 methyltransferase KMT2B/KMT2D can kill dormant CSCs, providing a theoretical basis for optimized cancer treatment [154]. The complex of the histone deacetylase inhibitor valproic acid (VPA) and copper (II) can synergistically induce the apoptosis of cancer stem cells [180]. In addition, 5-azacytidine (Aza), a DNA methyltransferase inhibitor, and its structural analog 5-aza-2-deoxycytidine (Aza-dC) have shown considerable therapeutic efficacy against myelodysplastic syndrome and other hematological malignancies. These compounds work mainly through the breakdown of the epigenetic memory of cancer stem cells, which then undergo apoptosis or terminal differentiation [181]. Recently, many highly specific small-molecule inhibitors of epigenetic modification enzymes have emerged, especially those targeting the histone methyltransferase EZH2. These drugs can precisely control histone methylation levels, thereby effectively eliminating dormant CSCs and providing a new drug intervention strategy for the clinical treatment of malignant tumors [182].

### Exploration of combination therapy strategies

Combination therapy strategies have shown great potential for clinical translation in targeting dormant CSCs. For instance, the use of antiangiogenic agents and cytotoxic chemotherapy drugs can result in dual benefits in liver cancer. In addition, the CD13-targeting drug bestatin can be included in the standard treatment plan to reduce the number of dormant CSCs [91]. For breast cancer treatment, blockade of both the Src family kinase and MEK1/2 signaling pathways can increase the eradication efficiency of dormant CSCs [183]. When dendritic cell vaccines are used in combination with immune checkpoint inhibitors, synergistic immune activation effects result, effectively eradicating cancer stem cells and reducing the risk of recurrence [184]. In the treatment of glioblastoma, 3-bromo-2-oxopropanoate-1-propyl ester has a synergistic effect with carmustine. This combination improves the clearance of glioma stem cells by quickly depleting their energy reserves and blocking the DNA repair processes triggered by carmustine[185]. In the treatment of lung cancer, the combined application of photodynamic therapy with traditional chemotherapy and radiotherapy has shown a synergistic advantage. This strategy not only enhances the direct cytotoxic effects on tumor cells but also targets dormant CSCs through a multitargeted synergistic approach, thereby effectively reducing the risk of tumor recurrence and metastasis[186]. Notably, bone marrow-derived mesenchymal stem cells (MSCs) play a crucial role in the tumor dormancy regulatory network through exosome-mediated intercellular communication. Therefore, targeting exosomes derived from MSCs can prevent the survival of dormant CSCs and reduce bone metastasis [187]. The Notch, Hedgehog and Wnt/β-catenin signaling pathways have been identified as important regulators of osteosarcoma CSCs. Targeting these pathways may be a promising therapeutic strategy for improving the prognosis of osteosarcoma patients [128].

## Controversy and future prospects in the study of dormant CSCs

### Controversies in the study of dormant CSCs

In regard to dormant CSCs, fundamental scientific challenges still exist. Some researchers have suggested that cancer stem cells and disseminated tumor cells may serve as dormant progenitor cells. However, the molecular mechanisms that induce a dormant state are not well understood. The dormancy of cancer stem cells is controlled by a number of factors, including crucial intracellular signal transduction, the tumor microenvironment and epigenetic modifications. Moreover, the relationships among different regulatory systems for inactivation and activation during dormancy remain unclear [26, 40]. The interaction between dormant CSCs and the immune system is complex. On the one hand, immune surveillance can inhibit the biological activity of dormant CSCs; on the other hand, dormant CSCs can evade immune recognition by upregulating immunosuppressive molecules [62]. Although conventional radiotherapy and chemotherapy clearly suppress tumors, their ability to kill dormant CSCs remains debated [188]. In addition, many detection methods for dormant CSCs, including molecular imaging and biomarkers, have technical drawbacks, such as excessive insensitivity, limited specificity, and complex operating procedures, which often make them unreliable for accurate clinical diagnosis. Overall, developing an innovative detection technology system that has high sensitivity and clinical applicability to facilitate early diagnosis, monitor therapeutic efficacy and predict recurrence is highly important [189, 190].

### Frontier technologies in the study of dormant CSCs

The rapid development of new biotechnologies in recent years has contributed to the detailed study of the dormancy process of CSCs. For example, single-cell sequencing technology has been used to successfully differentiate important molecular differences between dormancy and a proliferative state by analyzing the transcriptomic profiles of individual CSCs [191]. Studies have shown that CSCs display distinct molecular markers during dormancy. In the context of colon cancer, the expression of p27 in dormant CSCs is elevated, which provides a molecular basis for the development of targeted therapies against dormant CSCs [72]_._ Moreover, gene editing techniques such as CRISPR-Cas9 offer powerful tools for analyzing tumor stem cell dormancy. By precisely editing key genes, their roles in dormancy regulation can be accurately identified [68]. The study of dormant CSCs has greatly benefited from organoid culture technology, which can accurately mimic the in vivo microenvironment and reproduce the important biological processes of tumor stem cell growth, quiescence, and differentiation. Notably, the abovementioned organoid models serve as powerful experimental systems for evaluating antitumor drugs with the goal of personalizing treatment [192].

### Potential therapeutic targets for dormant CSCs

A series of possible targets, such as kinase inhibitors and cell cycle regulators (e.g., p27 and p21), that contribute to tumor stem cell dormancy may serve as potential therapeutic targets. By accurately controlling the expression of p27 and other molecules, the balance between dormancy and proliferation can be disrupted to allow quiescent CSCs to re-enter a proliferative state, thereby enhancing the effectiveness of traditional chemotherapy drugs and providing a theoretical basis for the development of new antitumor treatments [71, 72]. In the bone marrow microenvironment, mesenchymal stem cells communicate with cancer stem cells by secreting specific cytokines and exosome components to maintain their dormant state. Blocking the cytokines or exosomes related to dormancy-promoting factors can effectively disturb the system regulating tumor stem cell dormancy [187]. Recently, several new specific markers of dormant CSCs, such as p27, CD13, QSOX1, Survivin, GPD1 and BEX2, have been identified, which offer hope for targeted therapy [68, 73].

## Conclusion

It is important to incorporate different omics technologies, such as transcriptomics, proteomics, and metabolomics, in basic research to obtain a full understanding of the molecular regulatory networks of cancer stem cells in different physiological states, which may provide a theoretical basis for the identification of novel therapeutic targets and diagnostic biomarkers [61]. Optimization of molecular imaging technologies and enhancement of liquid biopsy methods can enable noninvasive tracking of dormant CSCs [193]. With an in-depth understanding of the biological characteristics of dormant CSCs, the combination of targeted therapy, immunotherapy and chemotherapy is expected to help establish personalized and accurate treatment models [183].

## Data Availability

Data sharing is not applicable in this article as no new data was created or analyzed in this study.
